# Expression and clinical significance of the proliferation marker minichromosome maintenance protein 2 (Mcm2) in diffuse astrocytomas WHO grade II

**DOI:** 10.1186/1746-1596-8-67

**Published:** 2013-04-24

**Authors:** Tove Lind-Landström, Rosilin K Varughese, Stein Sundstrøm, Sverre H Torp

**Affiliations:** 1 Department of Laboratory Medicine, Children’s and Women’s Health, Faculty of Medicine, Norwegian University of Science and Technology (NTNU), Trondheim, Norway; 2 Department of Oncology, St. Olavs Hospital, University Hospital of Trondheim, Trondheim, Norway; 3 Department of Pathology and Medical Genetics, St. Olavs Hospital, Trondheim, NO-7006, Norway

**Keywords:** Brain tumours, Diagnosis, Gliomas, Immunohistochemistry, Ki67/MIB-1, Prognosis, Survival

## Abstract

**Background:**

The WHO classification system for astrocytomas is not considered optimal, mainly because of the subjective assessment of the histopathological features. Few prognostic variables have been found that stratify the risk of clinical progression in patients with grade II astrocytoma. For that reason there is a continuous search for biomarkers that can improve the histopathological diagnosis and prognostication of these tumours.

**Aim:**

This study was designed to investigate the prognostic significance of the proliferative marker Mcm2 (minichromosome maintenance protein 2) in diffuse astrocytomas WHO grade II and correlate the findings with histopathology, mitoses, and Ki67/MIB-1 immunostaining.

**Method:**

61 patients with histologically verified grade II astrocytoma (WHO 2007) were investigated. Paraffin sections were immunostained with anti-Mcm2, and the Mcm2 proliferative index (PI) was determined as the percentage of immunoreactive tumour cell nuclei.

**Results:**

Mcm2 PI was not associated with any histopathological features but correlated significantly with mitotic count and Ki67/MIB-1 PI (p<0.05). In the survival analyses Mcm2 showed trends to poorer survival, however, statistical significance was not achieved in the univariate analyses (p>0.05).

**Conclusions:**

In our hands Mcm2 immunostaining has no advantage over Ki67/MIB-1 in the evaluation of grade II astrocytomas. Larger studies are needed to fully clarify the prognostic role of this biomarker.

**Virtual slides:**

The virtual slide(s) for this article can be found here: http://www.diagnosticpathology.diagnomx.eu/vs/1715002791944037

## Background

The histopathological criteria for the diagnosis of astrocytic tumours, are given by the World Health Organization (WHO) [1]. This classification system is not optimal, partly due to subjective assessment of the histopathological features. Novel biomarkers are therefore warranted to improve diagnostic and prognostic accuracy in such a way that the clinician can work out potentially more effective treatment plans. Supplemental immunohistochemical and molecular diagnostic analyses are gradually being employed in the diagnosis and evaluation of astrocytomas. Relevant markers are for instance alterations in pathways of members of the epidermal growth factor receptor (EGFR) family, methylation status of the O-6 methyl-guanine-DNA methyltransferase (MGMT) gene, isocitrate dehydrogenase-1 (IDH1) mutations, and various proliferation markers [2-5].

Regarding proliferation, it is currently accepted that the growth rate of a tumour is closely linked to its biological behaviour. Determination of a tumour’s proliferative activity has therefore gained much interest, also for brain tumours [6-9]. This has traditionally been evaluated by counting the number of mitoses. This method still plays a fundamental role in various histological grading schemes, including human astrocytomas [10]. It is, however, encumbered with several disadvantages including the subjective assessment of mitotic figures and confusion with pycnotic cells resulting in considerable interobserver variation [7].

Due to these limitations, immunohistochemical determination of proliferative activity has come into daily routine. The most commonly used antibody is Ki67/MIB-1, which is an antibody directed against an antigen expressed during all active phases of the cell cycle [11]. However, due to a great spread of proliferation indices between different malignancy grades of astrocytic tumours, it is difficult to establish definitive thresholds values for prognostic and diagnostic purposes [9,12,13]. Nevertheless, most studies show that this marker is associated with both tumour grade and prognosis [9].

There is a continuous search for more specific markers, and the proliferative-associated minichromosome maintenance proteins 2–7 (Mcm 2–7) appear promising. They constitute an important role in regulating cell proliferation through recruiting the replication machinery. Up-regulation of Mcm expression has been shown in proliferating cells, indicating potential as a prognostic marker of malignancy [14]. In fact, it is considered that Mcm-proteins are more specific than that of Ki67/MIB-1 [15]. Studies on Mcm2 in gliomas are rare. As an example, in a study on oligodendrogliomas Mcm2 proliferative index (PI) showed good correlation with mitotic index, Ki67/MIB-1 PI, and survival [16]. Further, in meningiomas it was related to increased risk of recurrence [17].

The aim of this study was to investigate the prognostic role of Mcm2 expression in a series of diffuse astrocytomas WHO grade II and to correlate it with histopathological features, mitoses, and Ki67/MIB-1 immunostaining.

## Material and methods

This study consists of a series of primary intracranial diffuse astrocytomas WHO grade II in adults (age > 16 years) consecutively operated at St. Olavs Hospital, Trondheim, Norway, in the time period 1987 to 2007. Originally the study population constituted a cohort of 109 patients [18,19]. Due to lack of archive material only 61 cases were available for further immunohistochemical analyses. Patients were collected through search in the electronic database at the pathology department.

Information about age, gender, tumour location, treatment, symptoms, and performance score was retrieved from both paper and electronical files at the university hospital and local hospitals. The cause and date of death were recorded from the Norwegian Death Registry.

All the routine stained sections were revised, and the diagnosis was adjusted to the WHO 2007 criteria. Histopathological findings and Ki67/MIB-1 immunostaining have been published previously (Table 1) [18,19]. Formalin-fixed and paraffin-embedded sections (61 of the original 109 cases) underwent immunohistochemical analyses using an automatized immunohistostainer (Dako Techmate 500) with a standard avidin-biotin-peroxidase technique. The mouse monoclonal anti-Mcm2 (NCL-MCM2, Novocastra) was applied with a dilution 1:25. The sections were developed with diaminobenzidine and counterstained with haematoxylin. Human tonsils served as positive controls. In the negative controls the primary antibody was omitted. Microscopic areas with highest labelling intensity were chosen for calculations. In each case either at least 1000 tumour cell nuclei were counted or three high power fields (HPFs) were examined using an eye-grid. The PI was defined as the percentage of immunoreactive tumour cell nuclei.

**Table 1:** Histological features

***Histological features***	***No. of cases***	***%***
	***(Total no.: 61)***	
Subtypes		
Fibrillary	55	90.2
Gemistocytic	5	8.2
Protoplasmic	1	1.6
Cell density		
Low	16	26.2
Moderate	40	65.6
High	5	8.2
Atypia		
Slight	24	39.3
Moderate	36	59.0
Severe	1	1.6
Apoptoses	27	44.3
Mitoses		
None	41	67.2
One	14	23.0
Two	4	6.6
Three	2	3.3
Rosenthal fibres	2	3.3
Eosinophilic granular bodies	0	0.0
Microcysts	21	34.4
Myxoid matrix	7	11.5
Microcalcification	3	4.9
Perivascular lymphocytic infiltration	11	18.0
Secondary structures	40	65.6
Subpial	12	19.7
Satellitosis	41	67.2
Angiocentric growth	13	21.3

The SPSS statistics package 17.0 was used for statistical analyses. Survival curves were calculated according to the Kaplan-Meier method, and differences in survival were tested for statistical significance using the log-rank test and univariate Cox regression. Spearman’s rank correlation was used for investigation of relationship between the proliferation markers. For analyses of correlation between histological features and Mcm2 Mann–Whitney *U*-test was used. The kappa statistic was used to evaluate the interobserver variation.

The Regional Committee for Medical Ethics approved the study, and the study protocol adhered to guidelines by Helsinki Convention.

## Results

The median age of the 61 astrocytoma patients was 40 years with range 19–74 years. Gender distribution was 24 females and 37 males (ratio 1:1.5). Thirty-eight (62.3%) patients died during the study period and 45 (73.8%) had relapse recorded. Histological and clinical characteristics have been previously reported [18,19].

**Figure 1:**
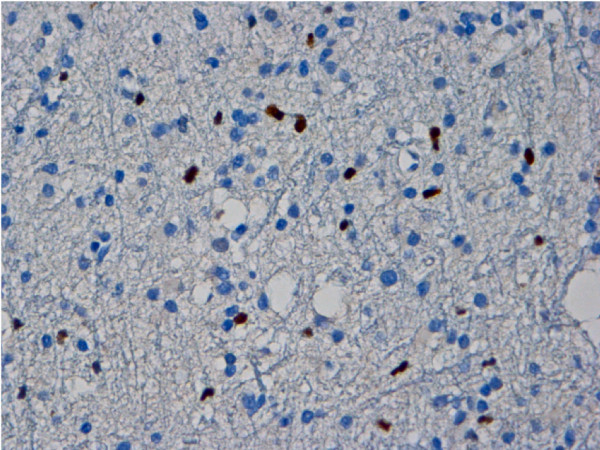
Image of a Mcm2 immunostaining of a grade II astrocytomas with positive neoplastic astrocytic cell nuclei (dark) (40× objective).

**Figure 2:**
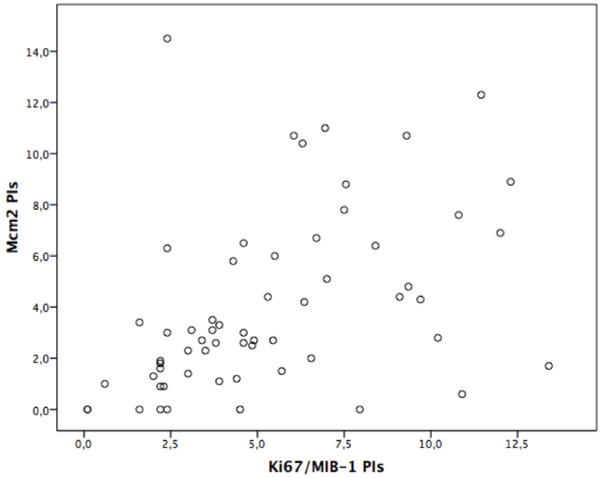
Scatterplot of Ki67/MIB-1 PIs versus Mcm2 PIs.

The Ki67/MIB-1 and Mcm2 immunostaining revealed distinct positive tumour cell nuclei heterogeneously distributed within the tumour tissue (Figure 1). Glia cells, neurons and leptomeninges were not immunoreactive. The median values of Ki67/MIB-1 PI and Mcm2 PI were 4.6% (range 0.1-13.4) and 2.8% (range 0–14.5), respectively (Table 2). Due to lack of material Ki67/MIB-1 immunostaining was performed on 60 out of 61 cases. Rank correlation showed significant correlation between PIs of Ki67/MIB-1 and Mcm2 immunostainings (p<0.01), also shown in a scatter plot (Figure 2). Significant correlation was also found between Mcm2 and mitotic count (p = 0.026) (Table 3). Kappa statistic gave a score of 0.6 indicating moderate agreement of interobserver variation.

**Table 2:** Descriptive statistics for and Ki67/MIB-1 PI and Mcm2 PI

	***Ki67/MIB-1 PI *[**[13]**]**	***Mcm2 PI***
No of cases	60	61
Median	4.6%	2.8%
Range	0.1-13.4%	0-14.5%

**Table 3:** Relationship between proliferative markers

***Spearman’s rank correlation***	***p-values***
Mcm2 PI versus Ki67/MIB-1 PI	< 0.01
Mcm2 PI versus mitotic count	0.026

The frequency of histological features is shown in Table 1. Correlations between histological features and Mcm2 immunostaining revealed only statistical significance with mitoses (p = 0.032) (Table 4).

**Table 4:** Relations between Mcm2 PI and some histopathological features

***Variables***	***p-values***
Mcm2 PI and mitoses	0.032
Mcm2 PI and apoptoses	0.302
Mcm2 PI and cell density	0.974
Mcm2 PI and atypia	0.395
Mcm2 PI and microcysts	0.267
Mcm2 PI and secondary structures	0.952

**Figure 3:**
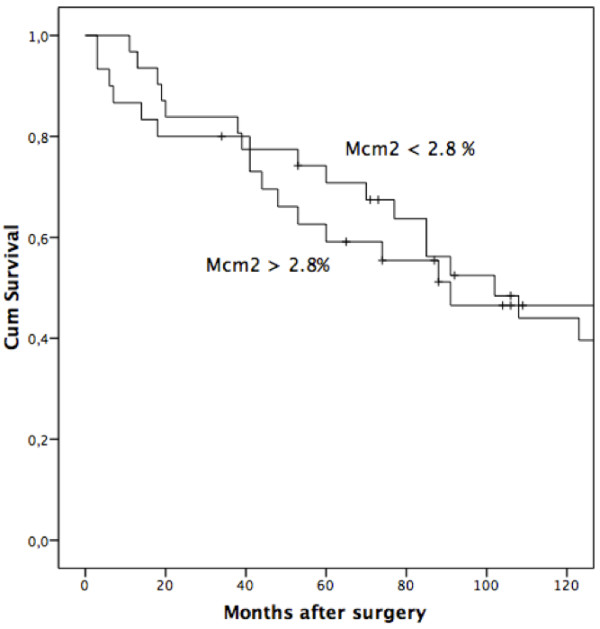
Kaplan-Meier survival curves for patients with different Mcm2 PIs (cut off at median value) (overall survival, log rank test p = 0.918).

Using the median value of Mcm2 PI as cut off, mean survival in the low proliferative group was 92 months compared with 78 months in the high proliferative group. Regarding overall survival and time to recurrence, no difference between the two groups was found (overall survival: log rank test (p = 0.918)) (time to recurrence: log rank test (p = 0.452)). Neither mitoses nor Ki67/MiB-1 immunostaining reached statistical significance in the survival analyses (mitosis: overall survival: log rank test (p = 0.092); time to recurrence: log rank test (p = 0.052)) (Ki67/MIB-1: overall survival: log rank test (p = 0.721); time to recurrence: log rank test (p = 0.285)). Kaplan-Meier survival curves for patients with high and low Mcm2 PIs are shown in Figure 3. Univariate Cox regression analyses with both overall survival and time to recurrence as time parameters were performed, and no significant difference in survival was found between these groups (Table 5). Even when the patients were stratified into two groups with PIs of Ki67/MIB-1 and Mcm2 PIs higher and lower than median values, no statistically difference in survival was achieved (p = 0.727).

**Table 5:** Prognostic factors: survival determined by univariate Cox regression analyses including both overall survival and time to recurrence

***Factors***	***p values***	***Significance***
*Overall survival*		
- Mcm2 PI	0.367	NS
- Ki67/MIB-1 PI	0.239	NS
- Mitotic count	0.099	NS
*Time to recurrence*		
- Mcm2 PI	0.407	NS
- Ki67/MIB-1 PI	0.516	NS
- Mitotic count	0.057	NS

## Discussion

Since the WHO classification system for human astrocytomas is not optimal, novel biomarkers are needed. Antibodies reactive against proliferation-associated antigens have therefore come into focus. This study was designed to evaluate the prognostic significance of the proliferation marker Mcm2 in a series of diffuse astrocytoma WHO grade II with thorough follow-up, and to compare its expression with histopathological features, mitoses, as well as Ki67/MIB-1 immunostaining. We found that Mcm2 expression correlated well with mitotic counts and Ki67/MIB-1 PIs but was not associated with any specific histopathology. Higher Mcm-2 PIs showed a trend to poorer survival, but statistical significance was not achieved.

The positive correlations between mitotic counts, Mcm2 PIs, and Ki67/MIB-1 PIs found in this study are in accordance with studies on other human tumours [16,20-22]. It seems, however, that the range of Mcm2 PIs varies between types of neoplasms. In our study Mcm2 PIs were lower than Ki67/MIB-1 PIs and comparable with those in pilocytic astrocytomas, whereas in oligodendrogliomas the Mcm2 indices were higher [16,20]. In colorectal cancer Mcm2 was shown to be a stronger indicator of proliferative cells than Ki67/MIB-1 [23]. The reasons for these various findings are not obvious, but may be related to different tumour types, malignancy grades, counting procedures, type of antibody, and antigen preservation [8,24]. Further, the immunoprofile of cycling tumour cells may vary. Mcm2 has been shown to stain cell-cycle initiation and continues to be expressed throughout the cycle including cells leaving G0 to enter early G1 [25]. In comparison, Ki67/MIB-1 immunoreactivity occurs during all active phases of the cell cycle except G0 [26,27]. Mcm2 may therefore be a biomarker of cells with replication potential (licensed to cycle), and suggestions on potential as a pre-cancer marker have been made [28].

Our survival analyses did not demonstrate any significant prognostic value of Mcm2. Nevertheless, a difference in survival of 14 months between the patients with high and low Mcm2 PIs indicates a promising trend. Actually, there is scarce knowledge about the value of Mcm2 in low-grade astrocytomas, so further studies are highly desired to fully establish its clinical significance in these tumours. Regarding other neoplasms, the findings are diverging. In a study on oligodendrogliomas with both low- and high-grade tumours positive association with survival was found [16]. In meningiomas high Mcm2 PI was associated with early recurrence [17]. On the contrary, no correlation to survival was established in a series of pilocytic astrocytomas [20].

Beyond the correlation with mitoses, Mcm2 was not associated with any other histological features related to malignancy in our series of astrocytomas, such as cell density, apoptoses, and atypia. In contrast, in a previous study Ki67/MIB-1 showed positive correlation with apoptoses, cellularity, and atypia [14]. We have no obvious reasons for this difference, but it may be related to lower values of Mcm2 in grade II due to earlier stage in the gliomagenesis.

Due to a limited number of patients and the well-known heterogeneity of astrocytic tumours, our data must be interpreted with discretion. The wide range of Mcm2 PIs points to possible overlap of values between astrocytomas of various malignancy grades comparable with that of Ki67/MIB-1 immunostaining [9]. It seems as if this phenomenon is a rather typical trait for proliferation markers reducing their utility in the routine diagnostics. Additionally, immunohistochemistry has its intrinsic adversity, such as the definition of immunoreactive cells (non-neoplastic and neoplastic cells, threshold values etc.), and several studies have poor definition of the quantitation procedures or reproducibility [7,8,29,30]. On the other hand, reliable follow-up data, combination of two proliferation markers, and the attempt to compensate for intraobserver reliability through Kappa statistic, are some of the strengths of this study.

In conclusion, Mcm2 immunostaining correlates well with mitotic activity and Ki67/MIB-1 expression. However, none of these were shown not to have any significant influence on patient outcome, even if the markers were combined. Accordingly, Mcm2 does not seem to have any advantages over Ki67/MIB-1 in the evaluation of the prognosis of grade II astrocytomas. However, larger studies are necessary to clarify the definite role of Mcm2 in these tumours.

## Competing interests

The authors report no conflicts of interest.

## Authors’ contributions

TLL carried out the data collection, analyses and interpretations, statistical analyses, and wrote the manuscript. RKV and SS critically read and edited the manuscript and assisted in the statistical analyses. SHT developed the study’s hypotheses and its design, and helped to draft the manuscript. All authors have read and approved the final manuscript.
